# Editorial: Coarse-Grained Models of Nucleic Acids and Their Applications

**DOI:** 10.3389/fmolb.2022.842049

**Published:** 2022-01-24

**Authors:** Maciej Maciejczyk

**Affiliations:** Department of Physics and Biophysics, University of Warmia and Mazury in Olsztyn, Olsztyn, Poland

**Keywords:** coarse-grained models, Nucleic acids (DNA and RNA), RNA folding, DNA repair, DNA transcription, RNA structure prediction, RNA pseudoknot

The art of molecular simulations of biological systems is related to the development and/or application of the best possible approximations of physical processes of interest. However, the large time- and size-scales of biological systems and processes preclude us from applying detailed quantum-mechanical methods or even empirical all-atom force field with molecular dynamics. Therefore coarse-grained methods, in which groups of atoms are represented by a smaller number of interaction centers, have been extensively developed in the last 30 years. The most obvious and important application of coarse-grained models is prediction of 3D structure of biopolymers. Physics-based coarse-grained methods not only are useful for structure modeling but also enable us to model the dynamics of the systems studied and to understand the principles of structure formation and dynamics. Initially, most of the laboratories working on coarse-grained models focused on the famous protein folding problem and therefore developed coarse-grained models of proteins. These models competed with other approaches with varying success in the Community Wide Experiments on the Critical Assessment of Techniques for Protein Structure Prediction (CASP). In the last 10 years, one can observe also a growing number of coarse-grained models of nucleic acids, which are mostly aimed at the prediction of RNA structure—the problem, which in many aspects is even more challenging than the protein structure prediction. In general coarse-grained models can be divided into two categories: physics-based and knowledge-based. Some models also used mixed physics- and knowledge-based approaches.

The mini-review of Li and Chen briefly describes the ideas, construction and performance of 12 models developed for the prediction of three-dimensional structure and thermodynamics of RNA. Almost all of the selected models utilize the knowledge-based approach, with the exception of oxRNA, which parametrization relies on thermodynamic data. In the mini-review, authors compare the performance of three models for which the source code is publicly available: namely iFoldRNA, SimRNA, and IsRNA1. The models are tested using 130 structures with lengths varying from 40 to 161 nucleotides. The authors compare structures predicted to the experimental ones using only a single parameter—RMSD. The average/median RMSD for all models is relatively high (around 1 nm) and according to this parameter the order of models is: IsRNA1, SimRNA, and iFoldRNA. This level of prediction is generally considered as practically useful as it should correctly predict positions of helices and general 3D structure. The computational efficiency of all models is comparable and it should not be a problem even for long RNA chains, for which the bigger issue might be efficient passing of large energy barriers, which in turn might require very long REMD simulations. In the final section, authors postulate three developments which should improve the quality of prediction: the energy function, improved sampling algorithms and using additional experimental data of: SAXS, cryo-EM, FRET, chemical cross-linking and footprinting.

The folding process and stability of RNA is usually investigated in pure explicit or implicit solvent. In reality, macromolecules exist in a crowded environment of the living cell which can influence their folding and stability. In the publication of Feng et al. the influence of spatial confinement on the stability of RNA pseudoknot is investigated. The authors performed folding simulations of mouse mammary tumor virus (MMTV) pseudoknot in three different spherical cavities over a wide range of Na+ ions concentrations using the coarse-grained model. The authors conclude that spatial confinement of RNA chain weakens the dependence of the compactness of the macromolecule on ionic concentration and temperature. Also the thermal stability of the pseudoknot is enhanced due to spatial confinement, especially at low salt concentrations. Authors also observe that number of unfolding pathways depends on both spatial confinement and Na+ ions concentration. They conclude that spatial confinement at high salt concentrations leads to an increased number of major unfolding pathways. These are important findings which expand our knowledge about influence of molecular crowd on the stability and thermodynamics of biopolymers.

Besides the RNA folding problem, coarse-grained models of nucleic acids are also very useful in the description of long time-scale processes, which details are inaccessible for both experimental methods and all-atom molecular dynamics simulations. The publication of Shino and Takada provides detailed insight into crucial steps of the transcription process. They investigate the switch between three consecutive states of the preinitiation complex (PIC) of polymerase II (Pol II) bound to DNA, namely: closed complex with all complementary bases of DNA paired (CC), open complex with small ∼6 bp bubble (OC) and initially transcribing complex with bubble size increased to ∼13 bp (ITC). Authors use coarse-grained models for the description of both protein complex (AICG2+ model) and DNA (3SPN.2 model) and apply back mapping algorithms to obtain all-atom structures from coarse-grained simulations. The most important observation is the presence of two intermediate states on trajectories leading from OC to ITC state. One of these states is caused by blockage of fairy well-conserved motif of Pol II called fork loop 1. The authors postulate that fork loop 1 plays a very important role as the gatekeeper, which can only be passed by the template strand and blocks the non-template strand of DNA. This is very important insight into the transcription process, which currently cannot be achieved by experimental or all-atom MD methods.

In the final publication of the Research Topic, Xie et al. tackle very important problem of DNA repair process performed by Uracil-DNA Glycosylase (UDG) enzyme. The enzyme scans DNA for the presence of uracil bases and binds closely to the uracil and then cuts it out of the DNA backbone. Authors use implicit solvent Poisson-Boltzmann model to determine forces acting between UDG and DNA. They conclude that although as expected forces are attractive over a wide range of distances between UDG and DNA there is repulsion between the UDG pocket and the uracil base. This study sheds light on the nature of electrostatic forces, which steers substrates towards each other in this important biophysical process.

We believe that our *Coarse-Grained Models of Nucleic Acids and Their Applications* research topic demonstrates the importance of coarse-grained models as nowadays they seem to be methods of choice for the description of long time-scale processes of macromolecular systems.

